# (Retrospective.) Mr. Charles Mansfield Clarke on Those Diseases of Females Attended by Discharges

**Published:** 1823-12-01

**Authors:** 


(RETROSPECTIVE.)

*Observations on those Diseases of Females which are attended by Discharges. Illustrated by Copper Plates.* By CHARLES MANSFIELD CLARKE, Esq. &c. Part I. on Mucous Discharges.

OUR readers are aware that in the second and third volume of the present series we gave an extended analytical review of the *second part* of Mr. Clarke's valuable work.* We have

---

* See Vol. II. p. 757—and Vol. III. p. 77.

received numerous letters requesting that we would render our review more complete by reverting to the *first part*, and thus enable the holders of the present series to consult the whole, as far at least as a review can serve the purpose of reference. We have reason to know that our Journal circulates widely where the original work has never gone, and never can go—we deem it therefore an object of some consequence to diffuse valuable matter whether recent or retrospective, among the more remote circles of medical society.*

The diseases of the sexual organs in females are certainly less known and understood by practitioners, than any other class of complaints; though they are generally distressing, and often fatal in their consequences, to those who labour under them. It is in vain for the student or practitioner to search the systems of physic or surgery for information on this head; and it requires much time, labour and expense to explore the periodical works, and detached collections and transactions of societies, for scattered observations on the diseases in questions. Mr. Clarke, therefore, has conferred a great benefit on the profession at large by the course of labours he has pursued, and is pursuing, in this novel field of enquiry.

The *anatomy* of the female organs of generation is, or ought to be, well known, and therefore Mr. C. swells not his work with what should be learnt in the dissecting room. The *discharges* from these parts, though all issuing from the os externum, spring originally from various sources, and are of different kinds. 1st. From the internal surface of the uterus and Fallopian tubes. 2d. The inner membrane of the vagina. 3d. The lacunæ about the os externum. 4th. The mucous membrane of the urethra.

1. The secretions from the uterus are, α. The menstrual secretion. β. The secretion from the mucous membrane of the same. γ. The glandular secretion from the cervix of the uterus.

α. Every person knows, that the menstrual fluid is not an effusion of blood, but a *blood-coloured secretion* from the uterus. It may, however, be remarked, that when the determination of blood to the uterine vessels is increased beyond a certain point, the orifices of the vessels give way, and blood is mixed with the secreted fluid.

---

* This article was originally drawn up for one of the monthly numbers of the "Medico-Chirurgical Journal," by the present reviewer; but that journal fell, having only reached a circulation of 160 copies! It may, therefore, be said to be comparatively unknown to the profession—*Rev.*

"If in consequence of this determination, inflammation takes place, then coagulable lymph is thrown out, and the secretion is diminished till the lamina of coagulating lymph is separated."

β. The *mucous secretion* is permanent through life, except during pregnancy. It resembles the uncoagulated white of an egg, and consists, like mucus in other parts, of albumen and soda. Its use is simply that of lubrication.

γ. The *glandular secretion* from the cervix uteri contains less water than any other mucus in the body; is semitransparent; tenacious like birdlime, and only secreted during pregnancy, especially in the first months of utero-gestation; its use is supposed to be, to prevent the ovum from escaping in its early state.

2. *Vaginal secretion.*—This mucus is generally thinner than either of the former. It is generally in greater quantity where the rugæ of the vagina are most obliterated, as in women of lax fibre—who have borne children, and who are grown old. In the latter, the rugæ have entirely disappeared.

3. *Secretion from the lacunæ of the vestibulum.*—These openings pour out a glutinous mucus which has a peculiar odour.

4. *Secretion from mucous membrane of the urethra.*—This requires no explanation.

### Chap. II. Profluvium Vaginale.

It is not of much consequence whether we denominate this discharge "γυναιχεια λευκα" with Hippocrates—"Fluor muliebris" with Sydenham—"Fluor albus" with Mead—"Leucorrhœa" with Cullen, or "Sexual weakness" with Hamilton, provided we can discriminate its causes, and treat it successfully. There are objections, however, to most of the foregoing appellations, particularly those which characterize the disease as exclusively the offspring of *debility*. The author's is probably the best, and his definition is clear.

"A discharge of a fluid from the vagina, varying in its consistence, quantity, and colour; either produced by weakness of the constitution, or by a change in the structure, position, or actions of the neighbouring parts, such change being the effect of natural or morbid causes." 31.

It is important to inquire into these causes; if the discharge be the effect of mere weakness, tonics will be required; if from a tumor, its removal or support. If from inflammatory action, tonics will evidently be injurious before the topical determination is taken off. In many cases, it would be as injurious to restrain vaginal discharges, as the natural salivation in a child during dentition. (*Vide our Reviews of Dr. Pary and Dr. John Clarke.*)

The discharges from the vagina may be classed as follow: viz. 1. Transparent mucous discharge. 2. White mucous ditto. 3. Watery discharge. 4. Purulent discharge. 5. Sanguineous discharge.

The *first* is gelatinous, nearly transparent, and coagulable. The *second* is opake, perfectly white, resembling a mixture of starch and water without heat. The *third* resembles clear water, having no colour, and very little glutinous matter. Its quantity is generally much greater than that of any of the others. The *fourth* resembles pus secreted from the surface of an ulcer. The *fifth* is of course composed principally of blood. The form in which this last comes away is strictly to be attended to. When the quantity is considerable, and from large vessels, it is fluid. When the reverse, it coagulates, assuming the figure of the parts in which it lies. When it slowly spreads itself over the surface of a tumor, it comes away in coagulated circles, which may be seen by putting the coagula in water.

The white mucous and watery discharges are pathognomonic of some uterine diseases; the sanguineous belongs to none exclusively. Of all vaginal discharges, those of mucus are the most frequent, as they are produced by several complaints of the parts, and are also the effect of mere debility in weakly constitutions. 40.

Chap. III. This is occupied with a few general observations on sexual diseases, and on the necessity of examination *par vaginam*. The *latter* is strongly insisted on by Mr. C. though we much fear that "the general disinclination of practitioners to make an examination," will continue as long as female delicacy exists. We agree with our author, however, that the examination should be urged with firmness, whenever the case is doubtful in its nature.

"It is notorious, that many practitioners prescribe for complaints of these organs (and Mr. C. might have added many other organs) from a mere history of the symptoms given by the patient."

The fallacy of these descriptions demonstrates the futility and even injury arising from acting on such vague information.

Chap. IV. On Sympathies.

There is very little new matter in this Chapter. We shall notice some of the sexual organ sympathies.

1. Disordered function in the *stomach*, renders the breasts flaccid, and diminishes the size of the gland itself. There is an exception to this during pregnancy, for very obvious reasons. 2. A blow on the testicles produces sickness at stomach. Functional derangement of the stomach renders the sexual passions dormant. 3. A diseased uterus produces sickness at stomach; this *last* causes *head-ache;* thus the uterus and head sympathize indirectly through the medium of the stomach. 4. The utero-gastric sympathy is the most common of all. In cancer uteri the stomach is always more or less affected with vomiting. When the uterus is ruptured, the *black vomit* comes on. 6. In amenorrhœa and dysmenorrhœa, the utero-gastric sympathy is equally remarkable. 7. The bladder and rectum sympathize with the uterus. 8. The lower extremities, in some cases, sympathize with the uterus, before and at the time of the menses. Pain in the lower limbs has been observed as a precursor of puerperal mania. 9. The *mind* is well known to sympathize strongly with the uterus. 10. The *brain* does the same as in furor uterinus, puerperal convulsions, and mania succeeding parturition.

### Chap. V. Diseases attended by mucous Discharges from the Vagina.

Some of these consist of the displacement of parts; as 1. Procidentia uteri. 2. Procidentia vesicæ. 3. P. vaginæ. 4. Inversio uteri.

*Procidentia uteri.*—In a healthy state of the parts, the os uteri is about an inch from the inner membrane of the vagina which forms a fold or reflection on its anterior surface. In procidentia uteri, this distance is gradually increased, according to the descent. The procidentia may be met with in all degrees till the uterus projects through the external parts, dragging with it the vagina, and forming a large tumor between the thighs of the woman. In this tumor will be contained the bladder, a portion of the rectum, and of the small intestines. Months, or even years, may elapse during this descent; for when the uterus descends to the perinæum, it there often rests, as on a cushion, for some time, the violence of the symptoms abating. The vagina, which is, of course, pushed down inverted before the womb, loses its florid colour from exposure to the air, and also its peculiar sexual irritability. The anterior part of the abdomen, from being convex, becomes flat. When the uterus and its appendages *only* have fallen out of the external parts, and before the other viscera have fallen into the inverted vagina, the tumor has a lengthened form, bearing a rude resemblance to the male organ, and giving origin to popular imposition. It may be detected by the orifice of the uterus having its *longest* diameter crosswise. As the viscera descend, the tumor becomes of a globular form. In this situation the parts contained in the tumor are liable to constriction and inflammation.

The immediate causes are, relaxation of the broad and round ligaments above; want of tone in the vagina below. Debility of the system is a predisposing or remote cause; "but the most common cause of procidentia uteri is the long-continued erect posture of the body at an early period after delivery, and in some cases after abortion." 69. At this time the womb weighs eight or ten times more than when unimpregnated, and descends by its own gravity.

> "The best practitioners direct their patients to remain in the recumbent posture upon a sofa, or on the outside of the bed; (where the advantages of horizontal posture and coolness are combined) till between the third and fourth weeks after delivery,"

when the uterus shall have regained its former diminution, and the ligaments and vagina their tone. A violent cough during the puerperal confinement may also produce this distressing event.

The symptoms of procidentia arise, some from dislocation of parts, others from sympathy. Pain in the back long precedes other symptoms; also pain in the groins, extending to the labia. There is a sense of fulness in the parts, and an increased discharge of transparent mucus from the vagina. The pain in the back is next described as a *dragging* pain, with a sense of bearing down. All these are relieved by the recumbent position. To these *strangury* is frequently added.

In procidentia uteri the symptoms arising from the utero-gastric sympathy are peculiarly distressing. The appetite fails; the stomach and bowels lose their tone; flatulence and borborigmi are troublesome; the spirits flag; employment becomes irksome, and life scarcely desirable.

The discharge varies much in quantity; but with this, and the impaired digestion, great debility arises. The menstrual fluid also is increased, and menorrhagia frequently attends. *Before* the external protrusion of the tumor, the discharge is greater than *after;* because the surface of the vagina ceases to secrete, when exposed permanently to the air. Hence, in the *former* state, the patient's debility is greater than in the *latter*. Patches of healthy looking ulceration soon attack the exposed vaginal surface, but seldom go deep; the os uteri is generally assailed by one of these.

Slight degrees of procidentia uteri can only be ascertained by anatomical knowledge and manual examination.

In this disease, a tumor hangs into the vagina, or out of the os externum. But every tumor is not a prolapsed uterus. The mark of discrimination is the os uteri at the lower part of the tumor.

The *recumbent* posture relieving the symptoms of incipient proc. uteri, it is evident, that the patient should be examined both in that and in the *erect* position, if we wish to avoid deception.

*Treatment.*—If nothing be done in the way of cure, the patient generally drags on a miserable existence, for a number of years, till destroyed by accident, or some other disease. Sometimes, though rarely, debility from the discharge, or inflammation of the contents of the tumor, puts an end to her sufferings. In its early stages, it is easily relieved, and the size of the support must be in proportion to the degree of dilatation of the vagina.

If, in this situation, conception should take place, a confinement for some weeks after parturition, in the horizontal posture, will often enable the parts to regain their tone, so as to render subsequent artificial assistance unnecessary. Where pregnancy does not take place, we must have recourse to art.

The curative intentions are, to increase the strength of the relaxed parts, (both topically and through the constitution) and to support the tumor. *Topically*, by cold, and by astringents. Cold water ought to be applied to the female parts; to the belly, and to the back, by means of a sponge, three or four times a day, the water being cool from the spring, or rendered colder by neutral salts. Cold water is also to be thrown into the vagina by means of a syringe. These and the horizontal position will be sufficient in slight cases. In more important cases, *astringents* are necessary. The sulphate of zinc is more useful in relaxations of the orifices of the mucous glands, than in want of tone in muscular fibres; it is also too irritating. The same observation is applicable to nitrate of silver and sulphate of copper. The superac. plumbi is inefficacious. Solutions of alum are more powerful and less irritating than either of the foregoing. A *combination* of astringents will sometimes succeed better than any single one. Alum and zinzi sulph. form a good combination in these cases. Among the vegetable astringents may be pointed out: Thea viridis; petala rosæ rubræ; cortex quercus; cortex granati; gallæ.

In aid of topical applications, tonics must be administered internally; especially the bitter tonics, as gentian, &c. with bark and sulphuric acid.

The bowels are to be nicely regulated; the extremes of constipation and diarrhœa being equally injurious. Rhubarb or senna is the best purgative in these cases, the resinous being irritating; the oily, nauseating; and the saline debilitating. The cold-bath, where the stomach is not much weakened, previously, will be found a valuable auxiliary. The salt-water bath is preferable to the fresh. Where the former cannot be procured, the author recommends the patient to be rubbed, on coming out of the bath, with a coarse towel, which has been wrung out of a solution of salt in water, and dried at the fire.

The recumbent posture on a hard mattress or sofa, is to be enjoined as much as possible in every case of proc. uteri, and the room of the patient kept cool.

"The diet should be nutritious, and a moderate quantity of wine may be allowed; but the stomach and intestines should never be loaded, lest the weight in the pelvis should keep up the complaint." 103.

*Pessaries.*—Those made of box wood are preferred to all others by Mr. Clarke; for the majority of cases, a circular or oval one answers sufficiently well. The circular pessary can only be used where the disease has not made much progress, and where the tone of the vagina is not much impaired.

"It will seldom be safe to introduce a circular pessary, the diameter of which exceeds too inches and a half; below this size, there can be no impropriety in using it; for it is certainly less liable to be displaced than the oval pessary. It should be large enough to keep the situation in which it is placed, else it will slip away; but it should not be so large as to distress the woman, or to injure the parts by its pressure."

Whatever may be the shape of the instrument employed as a support, it should be occasionally removed for the purpose of cleaning it; lest the secretions attached to it become acrimonious. Occasionally, also, the pessary should be changed for one of a smaller size.

The oval pessary rests with its longest diameter *across* the vagina, neither interfering with the rectum nor urinary passages. It is best adapted to those cases in which the tone of the vagina is so much diminished as to make a *large* support necessary. Its longest diameter should not exceed three inches and three-quarters. All pessaries should be introduced with care, and placed as high up in the vagina as possible.

The reduction of procidentia uteri, after its protrusion externally, must depend upon circumstances, and demands a prudent caution. Particular care should be taken to ascertain whether *inflammation* has, at any time, attacked the internal parts of the tumor; for in this case the attempt at reduction may tear open adhesions, and produce fatal, or at least dangerous consequences. When it is thought advisable, however, to replace the tumor, the bladder and rectum are to be first emptied, and the patient so placed that the pelvis may be much higher than the shoulders.

"The practitioner is then to apply his fingers and thumb to the lower part of the tumor, where the os uteri is situated, and by a gentle pressure this is to be carried up into the centre of the tumor itself. This done, the same pressure is to be continued, and the parts are to be returned into their proper place in the pelvis."

A pessary is then to be applied, and the patient should continue in the recumbent posture for several hours.

### Chap. VIII. Procidentia Vesicæ.

This may happen at any period of life, but will be most likely to occur when the vagina is relaxed, as after childbirth. It is the posterior part of the bladder which descends; most of the patients seen by Mr. Clarke have laboured under violent coughs. It has some symptoms in common with proc. uteri; some peculiar to itself. The sense of bearing down is not to the same extent as in proc. uteri. The size of the tumor, and the uneasiness of the part, are increased when urine is contained in the bladder, and vice versâ. A mucous discharge sometimes attends. The *peculiar* symptom (not noticed by any former author) *is a pain referred to the navel, with a sense of tightness there.* This pain is greatest when the bladder is fullest, the uneasiness diminishing as the urine is evacuated, till it totally disappears. Purgatives give a *temporary*, and only a temporary relief to this symptom. The symptoms resulting from the utero-gastric sympathy, as in the former chapter, are here wanting. A distinctive mark also occurs on examination.

In procidentia uteri an opening is perceptible at the lower part of the tumor; this is not the case in procidentia vesicæ. If the *origin* of the tumor be traced, it will be felt lying between the os pubis before, and the uterus behind, and will be easily recognized as formed by a fluid. The cervix uteri is also altered in this disease. The anterior lip of the os uteri is dragged down and very much lengthened; so that in this altered state of the parts, the os uteri, instead of being felt in the centre of the pelvis, opens directly backwards, and lies in contact with the posterior part of the vagina.

"Procidentia vesicæ admits of remedy by the use of a support introduced into the vagina; and the hollow pessary, of a globular form before mentioned, is more serviceable than any other." 131.

Solutions of astringent substances should be frequently thrown into the vagina; particular care being taken to avoid straining, as every exertion of this kind must affect the displaced part, and may force away the instrument. The bowels should be kept in a relaxed state, that the fæces may pass without any great exertion. Fruit and vegetables may therefore be indulged in; and when medicines are prescribed, the mildest are to be preferred. The urinary bladder should never be allowed to be full.

### Chap. IX. Procidentia Vaginæ.

This term is meant to imply a relaxation of the posterior part of the vagina, so that this part is lower than the natural defined edge of the perinæum. From the habitual constipation of females, their false delicacy and sedentary lives, the lower part of the intestinal canal becomes so distended sometimes as to make the posterior part of the vagina approach nearer to the anterior part of the pelvis, and in this way the diameter of the vagina is much diminished. This extreme distension of the gut takes off its power of contraction, while the strength of the sphincter is increased by resistance: When, at length, by natural efforts or purgatives, the rectum is emptied, the pouch formed by it and the posterior part of the vagina continues so as to form procidentia vaginæ. It is sometimes produced by piles acting in the same manner as habitual costiveness. It produces no effect on the shape of the os uteri, and recedes a little, but never entirely, when the patient is in the horizontal position.

Very few symptoms are complained of, except some slight pain in the back, and a trifling discharge of transparent mucus.

In curing the disease the bowels are to be regulated, and the tone of the gut restored. A globular pessary gives support to the posterior part of the vagina and to the weakened rectum. Solutions of alum, in a decoction of oak bark, may be thrown into the vagina several times a day, or applied by means of a sponge. Cold water applied to the loins and to the external sexual parts, will also assist the recovery by imparting tone to the organs; but costiveness is to be most carefully guarded against. Castor oil, salts, senna, and such laxatives should be preferred, especially if there be piles, to the resinous class of purgatives.

### Chap. X. Inversio Uteri.

This complaint is the inversion of the cavity of the uterus, so that the fundus comes through the os uteri: consequently, that part which was formerly the inside of a cavity, becomes now the outside of a tumor, either inor projecting from the vagina. It is almost always the consequence of mismanagement of the placenta, and of course, in these times, a rare occurrence. Its immediate effects are hæmorrhage, faintness, and a sense of fulness in the vagina. When discovered early, it is easily reduced to its natural situation.

"This is to be effected by making pressure on the *lower part* only of the tumor, so as to make this part to be received into that above it: a continuance of the same pressing force will, in some cases, quickly reduce the tumor." 143.

If the placenta be adhering to the uterus, the latter should be reduced before any separation of the former be attempted.

This disease is occasionally met with in a chronic state, and accompanied by a mucous discharge. The symptoms resemble those of procidentia uteri. It is to be distinguished from polypus, by the os uteri encircling the latter; whereas, in inversion of the womb, the os uteri forms a part of the tumor itself. Moreover, the inverted uterus is sensible, the polypus devoid of feeling. From procidentia uteri it is distinguished by the want of an opening at the lower part; from procidentia vesicæ, by being much more resisting; by its permanence of size, and by the impossibility of finding any uterus behind it.

When it projects externally during the age of menstruation, the catamenia will be observed coming from the whole surface of the *lower part* of the tumor. Whilst the inverted uterus remains in the vagina, the discharge, excepting at the catamenial periods, will be of a mucous kind. When it protrudes, the secretion is checked by exposure to the air; but ulceration is apt to succeed, and the discharge, of course, to become purulent.

Where the uterus has been long inverted, and lies in the vagina, it will not be advisable to recommend any thing but mild astringent injections thrown into the vagina three or four times a day; pessaries are useless, as there is no room for them.

When the inversion is in an extreme degree, the uterus hanging out of the body and dragging down with it the vagina, the discharge being great, and debility increasing, the patient's existence is either rendered miserable or cut off entirely, if the disease be left to itself. Here, if astringent and absorbent applications produce any abatement of the discharge, they should be persevered in, rather than risk an operation. But when the menstruating age is passed, and the patient's comfort is destroyed by the disease, the discharge threatening her life, it may be adviseable to recommend an operation which has been successful, and from which the author knew a patient recover in her sixtieth year. How far the operation may be prudent during the period of menstruation, Mr. C. pretends not to decide. Ambrose Paré, with Mauriceau and another surgeon, cut away the womb of a woman 20 years of age, who survived; but died of pleurisy three months afterwards.

"A poor woman, 60 years of age, complained of a tumor which hung down from the external parts between her thighs, attended by a discharge of mucus and pus, so profuse in quantity as to make her exceedingly weak. Upon an examination of the tumor, it appeared to be an inverted uterus, the whole surface of which was in a state of ulceration. Above this tumor was the vagina, also inverted, having partial ulcerations upon it. The circumstances in the life of the patient obliged her to apply to the dispensary for relief, and her increasing weakness made her readily consent to an operation for the removal of the tumor, which was performed by Mr. Chevalier, surgeon to the Westminster General Dispensary. A ligature was applied round the contracted part of the tumor; that is, where the uterus ended and the vagina began. It was tightened daily, until about the eleventh or twelfth day, when the parts included in the ligature were absorbed, and the uterus fell off. During this time the patient complained of very little pain. Adhesions had taken place between the sides of the vagina, so as to prevent the exposure of the cavity of the abdomen, and the woman recovered." 152.

### Chap. XI. On mucous Discharges produced by increased determination of blood to the sexual organs.

*Hæmorrhoids.*—This disease is a dilatation of the hæmorrhoidal veins, and is more common to females than males. A discharge of mucus from the vagina is a very common accompanyment to the piles; since the internal iliac artery supplies both the hæmorrhoidal and vaginal blood vessels; and it will be found difficult to restrain this discharge whilst the hæmorrhoidal tumors continue. The treatment of these tumors depends on the accompanying symptoms. If in a plethoric habit, and accompanied with sanguineous discharges, occasionally, it will not often be very prudent to suppress these discharges, lest some other part of the system should suffer. If the profuseness of the discharge, however, should induce debility, astringents and pressure may be safely employed. Every body knows what kind of *laxatives* (for *purgatives* are injurious) is useful in hæmorrhoids. Cold may be applied to the tumor by means of a sponge, or powdered ice may be applied between folds of linen. If the piles are external and painful, pressure may be made to return them within the sphincter, when the pain will be alleviated.

When the piles are removed, the mucous discharge often remains from the vagina. It is then to be treated by cold lotions and astringents, as before recommended.

### Chap. XII. Ascarides in the Rectum.

As mucous discharges from the urethra in men, indicative of disease in the prostate gland and testicle, are sometimes mistaken for, and treated as, a morbid state of the urethra, so mucous discharges from the vagina have been considered as originating in disease of that passage or of the uterus, when the irritation arising from ascarides in the rectum has produced all the symptoms. These worms are endowed with a very rapid motion, and have a pointed extremity with which they probably irritate the sides of the rectum, occasioning an insufferable itching, which is worse than pain to bear. This causes an increased determination of blood to the parts, and an increased secretion from the neighbouring mucous membranes. Ascarides will often travel across the perineum from the anus to the vagina, and there occasion a similar irritation. It is not always easy to discriminate these cases, since both mucous discharge and pruritus attend many other complaints requiring opposite modes of treatment.

In children, a mucous discharge from the vagina is not very unfrequently the effect of ascarides in the rectum, or irritation in the gums.

As the rectum is the seat of ascaris, or thread-worm, the complaint may be cured by glysters, partly by mechanically washing out the gut; partly by being obnoxious to the ascarides. Solutions of bitter substances in water, as chamomile flower decoction, wormwood, or a mixture of aloes and milk, are useful in removing them. But a strong decoction of semen santonici is the most efficacious of all the injections in use; with this the rectum should be filled: But the quantity should not be so great as to produce much distention of the gut, as the latter will contract suddenly and expel the glyster, which acts with more certainty, if it remains for some time. This operation repeated for a few successive days will seldom fail to remove for a time, at least, the ascarides and the symptoms they produce.

Purgatives alone are of little service; but during the use of glysters, they are to be occasionally exhibited. Jalap, scammony, calomel are the best. The *symptomatic* discharge from the vagina will disappear with its cause.

### Chap. XIII. Carcinoma Recti.

The subjects of this and the preceding chapter are introduced by Mr. C. because they are accompanied by mucous discharges from the vagina. In carcinoma recti, the capacity of the canal being diminished, the passage of the fæces through it is impeded, and painful. In consequence of this impediment in the rectum, the colon becomes greatly distended in some cases, and vast fæcal and other accumulations are occasionally found after death. The disease has often been mistaken for a stricture of the rectum, and much mischief done by attempts to dilate it by bougies. The pain attending the complaint is of the lancinating kind, and being referred to the region of the uterus, has led to a supposition of the disease being there. This error, however, is not of much consequence, as the principle of treatment is the same in both cases.

In car. recti the pain is increased greatly during the passage of the fæces. In stricture the pain will be, by no means, so acute. In car. recti acute pain is also occasionally felt when no endeavour is made to expel. In stricture, the pain is only felt at this time, or for a short time afterwards. In carcinoma also, the constitution sympathizes with the parts more than in stricture. In this complaint, the hæmorrhoidal veins are apt to enlarge and to bleed; the latter circumstance retards the progress of the malady. In the latter stages of carcinoma recti, and probably in the earlier, the mesenteric glands are affected according to the testimonies of writers on morbid anatomy; this accounts for the rapid progress of emaciation. The *scirrhous* state, by proper management, may be kept stationary for a considerable time. When the parts become *cancerous*, the symptoms assume a formidable aspect, and the mucous discharge is converted into a purulent one. Occasionally in the ulcerated stage, a communication is formed between the rectum and vagina; the pain becomes more acute; the stomach sympathises much, and hectic fever sometimes supervenes.

*Treatment.*—This disease admits not of *cure*, because extirpation cannot be performed; palliation is all we can afford. Stimulating applications to promote absorption of such tumors are detrimental. So are adhesive plaisters, however innocent or sedative in their natures. Common soap plaister spread upon thin linen is the best. The parts should be defended from the cold, but not kept too warm. Motion in the parts should be avoided as much as possible. Leeches are serviceable, especially where the scirrhus is deep seated.

When the skin appears on the stretch from the subjacent tumor, leeches are dangerous.

There is an intermediate stage between scirrhus and open cancer, which may be termed the *scabbing* stage, during which the patient may live many years, with less inconvenience and pain, than in the latter part of the scirrhous stage. When the cuticle bursts, an oozing of serous matter escapes, forming, first, a film, and afterwards a thick crust, covering even a portion of the contiguous sound surface. As long as the scab remains attached, the patient is safer than when it falls off. During the formation of this scab, the surface may be sprinkled with some light absorbent powder, as starch and oxide of zinc; the part should also be exposed to the air. All possible care is to be taken that this scab be not disturbed. These observations can only apply to carcinoma mammæ, and are a digression here; no such intermediate state is found in carcinoma of internal parts.

In treating carcinomatous tumours, every thing which can stimulate the parts, or increase the force of circulation in them, is to be carefully avoided. Where there are strength of constitution and local vascular action, blood should be taken from the region of the sacrum by the scarificators or leeches, and the operation repeated *pro re natâ*. Great attention should be paid to the bowels, which are to be kept in a soluble state by expressed oils, sulphur, manna, senna, or salts. Animal food, and fermented or distilled liquors are injurious. Sexual intercourse, from the vicinity of the rectum to the vagina, should be avoided. If the bladder become irritable, the semicupium is useful. Opium should be avoided as long as possible. The mucous discharge should not be restrained by astringents, for obvious reasons; *tepid* water may be thrown up the vagina frequently, and the external parts washed with the same.

The attention of the practitioner being called to the existence of organic disease, an examination should be insisted on. Perhaps the disease may be out of reach, either by the rectum or vagina. In such cases, if astringent injections produce an augmentation of pain, it will be prudent to act as if organic disease were known to exist. Such conduct can do no harm.

### Carcinoma Uteri.

In the commencement of Carcinoma Recti and C. Uteri, the two diseases may be distinguished; but in the latter stages, the surrounding parts are drawn into disease in both cases, and both are equally fatal.

Scirrhous enlargements of the uterus will sometimes go to a great extent without ulceration. The hard tumor rising from the cervix uteri, and hard thickening of the cervix itself, both disposed to ulcerate, are the subjects of the present chapters.

Carcinoma uteri is much more common than C. recti, attacking principally middle-aged women, with symptoms not very violent at first, but gradually increasing. By C. uteri, Mr. Clarke means a tumor near to, or a thickening of, the cervix uteri, disposed to ulcerate. The disease at first attacks *only* the cervix; all other tumors, however hard in their texture, are of a different character, and have a different termination—they do not ulcerate. Real scirrhous tumors seldom become large. Two varieties of this disease are distinguishable at an early stage.—1st var. A firm tumor of a rounded form, springing from the surface of the cervix uteri, or imbedded in it, the other parts of the uterus (excepting a thickening of the parietes) being perfectly healthy. 2d var. Enlargement and induration of the whole cervix uteri, in structure carcinomatous. In the first variety, there will be some symptoms from mechanical pressure of the tumor on neighbouring parts. In the second variety, these will not take place; because the carcinomatous thickening will rarely acquire a sufficient size to produce them. In women of temperate habits, the disease may go on for a long time without occasioning much inconvenience. But in real carcinomatous tumors of the uterus, the progress is very rapid when any violence is offered them, as in labour. A sense of weight in the vagina is equivocal, as it is felt in all moveable tumors of the pelvis, when they become large. A mucous discharge is a very constant attendant, and checks the progress of the complaint, by unloading the vessels of the womb. This discharge is sometimes tinged with blood, at others, blood itself comes away in great quantities; during which, the tumor generally remains stationary. Profuse evacuations of this kind sometimes end in dropsy, which terminates in death. In C. uteri, if menstruation has not ceased, it becomes irregular and profuse. Dysury is of rare occurrence here; but strangury, from sympathy, is frequent, with mucous secretion from the bladder. The pain counterfeits that attending the passage of a stone from the kidney to the bladder, and is sometimes accompanied by urticaria, and cardialgia. If, in these cases, tonics, alkalies, bitters, aromatics, or particularly mercury, be given, the original disease will probably be increased; hence the necessity of an examination, or minute inquiry, when the foregoing symptoms are observed.

The os uteri, in these cases, generally appears larger than usual, so as to admit the point of the finger, which feels as if surrounded by a firm ring.

*Treatment.* If the system is plethoric, blood should be taken from the arm proportioned to the circumstances of the case. The same may be done from the hypogastrium, or loins, by leeches or cupping, repeated from time to time on any increase of uneasiness. Blood is more easily taken from the back than from the abdomen. Saline purgatives, largely diluted, should never be omitted. Phos. sodæ will often agree, when all others irritate the stomach. The secretion from the vagina should never be checked, but washed away occasionally by injections of *tepid* water, neither so hot as to increase the vascular action of the vessels; nor so cold as to stop the secretion: from 86° to 94°. Warm clothing, as determining the blood to the surface, is useful; and as lessening the chance of visceral inflammation from atmospheric changes. All local stimuli, sexual intercourse included, are improper. The antiphlogistic regimen, in all its strictness, to be pursued.

Urticaria being a frequent attendant, and as the *febrile* state of the system during this eruption *is detrimental* to the local affection, it should be paid attention to. The bowels should be emptied by a purgative of rhubarb, magnesia, compound spirit of ammonia, and mint water, succeeded by a light bitter infusion thrice a day; to which may be added some carbonate of soda, potash, or aromatic water. Soda water for common drink. Here vegetables disagree, and small quantities of light animal food may be allowed.

If discharges of blood be moderate, they should not be checked; if profuse, the recumbent posture, cold, and local astringents are proper. Preparations of iron are injurious, and always increase the pain and discharge. No cure can, of course, be expected.

### Chap. XVI. Polypus of the Uterus.

Polypus Uteri is an insensible tumor attached to the internal part of this viscus by a small neck. These tumors are very various in respect to size, shape, hardness, &c. The neck of the tumor, surrounded by the os uteri, is contracted, while its base dilates in the yielding vagina. The soft polypus is by no means so common as the hard kind.

*Symptoms.* First, a mucous discharge, in considerable quantity, tinged occasionally with blood, and often inducing great debility before the nature of the disease is suspected. Instead of mucus, coagula of blood are sometimes voided; at others, pieces of a ring like form, from obvious causes. Great fœtor sometimes takes place from the blood remaining in the vagina, inducing a belief that cancer exists; which opinion is strengthened by the sickness of stomach attending the disease. Sense of pressure and bearing down, with pain in the back and groin, are felt in this disease. If the tumor be large, it may press both on the rectum and meatus urinarius obstructing the evacuation of fæces and urine; but such instances are uncommon. Thus gastric irritability, increased secretion of mucus, hæmorrhage, and derangement of the digestive powers, all tend to produce great debility in this complaint. But the true character of the disease can only be ascertained by examination. The only diseases for which polypus may be mistaken, are inversio uteri, and the cauliflower excrescence. The history of the case, and the *insensibility* will distinguish it from the former. The irregularity of surface, origination from the substance of the os uteri, broad base, and watery discharge, attending cauliflower excrescence, will distinguish it from polypus. There is, however, a disease, consisting of a tumor which is insensible, unequal and ragged in surface, coming down from the cavity of the womb into the vagina, surrounded by the os uteri, but without a narrow neck, which may be taken for polypus. Upon an accurate examination, this tumor will be found to be made up of a number of irregular portions which lie parallel with each other. All the symptoms of polypus uteri attend this complaint, and it ultimately proves fatal. M. Herbiniaux, a surgeon at Brussels, has given a good description of this tumor.

Polypus uteri is only to be cured by an operation, which is the application of a ligature round its neck. The prognostic is, in general, favourable.

### Fleshy Tubercle of the Uterus.

This is a hard, whitish tumor, situated sometimes upon the surface of the uterus, between the muscular and peritoneal coat, sometimes projecting into the cavity of the uterus, and occasionally imbedded in its substance. There is sometimes more than one tumor; their forms various; commonly spherical or hemispherical.—When projecting into the cavity of the uterus, their surface is smooth.—When on the exterior of that organ, they are otherwise. These tumors are sometimes not larger than a pea; sometimes they weigh several pounds, and occupy a great part of the abdominal cavity. The fleshy tubercle has been mistaken for ovarian dropsy and pregnancy. The first is an error of little importance, as the symptoms in both are mechanical, and do not interfere with life, unless by pressing on contiguous parts. The fleshy tubercle of the uterus is much more resisting than the cyst of an ovarian dropsy. In the latter, fluctuation may be felt by striking the abdomen gently with the hand. From pregnancy, it is sure to be distinguished, after a few months, by the motion of the child in the former. It is not an uncommon disease—occurs in the married and unmarried, but rarely before the twentieth year. It has no disposition to ulcerate, or suppurate; but inflammation is apt to occur in the neighbouring parts. Nothing is known respecting its cause.

*Symptoms*. An increased discharge of transparent mucus sometimes attends. The earlier symptoms are frequent disposition to make water, and to empty the rectum: retroversio uteri, and suppression of urine may occur in this disease. Cramp in one or both legs; œdema of one or both feet. When the tumor becomes large, there may be difficulty of passing the fæces, and total inability of emptying the bladder; bearing down, not relieved by the horizontal posture. On examination, a large, hard, resisting tumor will be felt; but the os uteri will have undergone no change—will not gape as in carcinoma; nor will pain be complained of when the tumor is pressed upon. The constitution is seldom affected; and when it is so, it is merely from pressure. It is totally uninfluenced by medicines internally or externally applied. The occasional symptoms only can be attended to with any advantage.

The XVIIIth. On verrucæ, or warty tumors, arising from the vestibulum, contains nothing very new or very interesting.

The two last Chapters in this work, XXI. and XXII. are interesting.—Viz. On the transparent mucous discharge from the vagina, unaccompanied by any organic derangement of the sexual organs.

There are two distinct and dissimilar classes of these discharges:—one arising from increased action of the vessels of the parts; the other from debility, in which the former, indeed, may end. Those discharges are, however, much more frequently from debility than increased action.

### From increased Action.

Women of naturally plethoric habits, possessing great strength of constitution, are more liable to this than weaker women. Females, who, in the middle of life, indulge much in the pleasures of the table, especially in wine or spirits—whose habits of life are sedentary, and who take little exercise in the open air, are liable to become suddenly corpulent, and form too much blood, as may be seen by the pulse and superficial veins. These women are in reality weak, though apparently strong—they are easily fatigued, and devoid of energy. In many of these, a slow enlargement of the liver takes place, as may be ascertained by the hand, while little bile is secreted, and the stools are clayey or white, with much fœtor of putrescency. Constipation and obesity increase; while a vaginal discharge takes place, and the menstrual flow is augmented. Upon enquiry, giddiness and somnolency will be complained of, with head-ache, perhaps indistinct vision, or the appearance of sparks before the eyes. Many years may elapse in this way, without much apprehension of danger, the symptoms being occasionally relieved by a spontaneous hæmorrhage from the nose, or other natural or artificial evacuation; when, all at once, the woman shall be attacked with apoplexy, or some great internal hæmorrhage that quickly terminates the scene! Or she may gradually become dropsical, and die that way. The mucous discharge is, in these cases, probably an effort of Nature to relieve the vascular plethora of the system; and hence, when injudiciously checked, without substituting other evacuations, the violence of the symptoms is increased.

Mr. C. has examined the bodies of females dying during the prevalence of these symptoms, and has found the uterus somewhat, but not much enlarged; while the liver was often increased to twice its natural size, with an uniform density and yellowness, but no other particular morbid alteration of structure.

"The objects in the treatment of this case are, to unload the vessels, by removing at once a large quantity of blood; to prevent its too quick formation in future; to restore, if possible, the liver to a healthy state: afterwards to moderate the vaginal discharge, or to diminish the inconveniences attending its continuance; and, lastly, to lay down proper rules for the patient's conduct, in order to prevent a return of the symptoms."

To obviate local fulness, if such be present, cupping glasses between the shoulders; to the lower part of the abdomen; to the loins; to the region of the liver. If no such local symptoms be distinguishable, venesection; saline purgatives in small doses thrice a day; antiphlogistic regimen. Exercise is to be gradually increased; fermented and spirituous liquors to be prohibited; and if their use had been long continued, with gastric debility, spices, aromatic seeds, and ammonia, may supersede them. Until the plethoric state of the system be reduced, tepid water *alone* is to be injected into the vagina; afterwards weak solutions of sulphate of zinc. Local increased action of the vessels, as frequent sexual intercourse, may give rise to the disorder, and is to be obviated if possible. After local or specific inflammation of the vagina, with purulent secretion, leucorrhœa sometimes succeeds, and is very difficult of cure.

In the treatment of transparent mucous discharges from the vagina, produced by increased action of the vessels, local remedies will be principally required, consisting of abstractions of blood by leeches, scarificators to the lower parts of the abdomen, or to the back, repeated when necessary. The bowels to be kept open by mild purgatives. The food to be simple and light, avoiding spices and liquors. Sexual intercourse to be prohibited. Cold solutions to the parts, (liq. plumbi acet. c.) and afterwards, when the secretion continues from relaxation of the membranes, astringent washes.

The last chapter in the work, on transparent mucous Discharge depending upon Debility, is short but interesting. We shall give it nearly in the words of the author.

"That women, whose vagina has lost its tone, become liable to this disease, has been before remarked in that part of this work where some general observations upon the nature of the vaginal discharge were made. Whatever tends to produce debility of the system, may lay the foundation of this complaint; such as long diseases, profuse hæmorrhages, or anxiety of mind.

"Women who live in a moist atmosphere, who keep bad hours, who spend much of their time in bed, or who inhabit hot rooms, (being generally weak women, and having a relaxed vagina,) will be apt to be affected by the complaint.

"It sometimes arises in women who suckle their children for too long a time, and it will often subside spontaneously upon the child being weaned.

"The quantity of discharge, and also its consistence, is very various in different cases. It sometimes comes away in a liquid form; at other times it is ropy.

"A pain in the back attends many cases of the disease. This symptom is, however, frequently found where great debility of the system is present.

"The continued drain from the system increases the original weakness; and the quantity of blood remaining is by degrees so much reduced, that the surface of the body becomes every day paler, till at length the cutaneous vessels are completely emptied of their contents; and at this time the skin assumes an appearance resembling that of a dead body. The colour of the sebaceous glands of the skin is evident through the cuticle; so that to the paleness of the skin is superadded an appearance of yellowness, which is not the effect of absorption of bile, for the urine will be found clear and colourless, and the tunica sclerotica of the eye will retain its pearl-coloured appearance. The exact balance between the secreting arteries and the absorbents being destroyed, the cellular membrane becomes filled with fluid, and the integuments acquire a doughy look and feel. This fluid effused, pervades the cells of the cellular membrane throughout the body; the legs and feet swell towards night, and in the morning this swelling subsides, and the face becomes puffed; a shortness of breathing succeeds, which is increased by the horizontal posture, and is rendered most distressing when the patient is going up an ascent, or endeavours to read aloud. Violent palpitations of the heart occasionally give the woman great uneasiness; and this symptom sometimes increases to so considerable a degree, that the action of the heart may be heard by a by-stander. During the continuance of these palpitations, the patient becomes very faint, and often considers herself to be dying.

"The circulation in the extremities is very languid, and the hands and feet are almost always cold; the pulse is feeble, sometimes very quick. The digestive organs not only partake of the general debility, but have more than their proportion of weakness. The appetite for food is lost; the power of digestion is diminished; and, from the spontaneous changes which the food undergoes in its passage through the stomach and intestines, the patient becomes much annoyed by flatulence.

"Bile is secreted very irregularly, and sometimes this secretion is suspended. Costiveness is a general attendant on this state of disease. In the farther progress of the case, hectic fever comes on, the difficulty of breathing becomes more extreme, and the patient dies with the symptoms of water in the chest. Although these symptoms are of the most formidable kind, and threaten the life of the patient, they frequently yield to the employment of proper means, which must be directed with skill, pursued with energy, and continued with patience.

"The first care of the practitioner should be to remove, if possible, the causes of the disease. If the patient has been living in a moist unhealthy situation, she should be removed to one which is more dry and salubrious: without attention to this very important circumstance, all the resources of art will be useless.

"It has been stated, upon good authority, that the disease is frequent in Holland, and that it is epidemic in wet autumns. The author has repeatedly seen the complaint attended by the worst symptoms in women who live in damp situations, and in the crowded parts of London, in whom they have quickly disappeared upon a removal to a more healthy spot. The woman should not be permitted to breathe a confined air. When the weather permits, she should go out; and the apartments in which she lives and sleeps should be large and well ventilated. Her habits of life should be regular. She should rise betimes in the morning, and retire to rest early in the evening. If she is too weak to sit up the whole of the day, a sofa is to be preferred to a bed.

"Persons who are very weak are much disposed to sleep. This prevents that exhaustion of the powers of the frame which would otherwise take place. Exercise, proportioned to the strength and to the means of the patient, should be recommended: when the weather permits, it should be taken in the open air. Exercise in an easy carriage is preferable to walking, and that taken on horseback to both. The chamber-horse, or elastic plank, may be employed when exercise out of doors cannot be used.

"The food should be of the lightest kind, such as animal broths and jellies, vegetable jellies, bread properly fermented and well baked. It will be better that the patient should not eat solid animal food until the powers of the stomach are in some degree restored, lest fever should be excited by it. When the powers of the digestive organs become more vigorous, recourse may be had to animal food; which should be taken in small quantities, once only in the twenty-four hours, and in tbe middle of the day. Tender meats, and such as will not be likely to disagree with the stomach, should be selected. Wine, either pure or mixed with water, as may best suit the palate or the stomach, may be allowed in moderate quantities. All wines which have not undergone a complete fermentation will give occasion to flatulency, and are therefore improper.

"The medicinal treatment is to consist in the employment of means to invigorate the stomach and the constitution, and to restrain the vaginal discharge, which, as long as it continues, must increase the debility.

"If steel and the other metallic tonics are exhibited in the first instance, they will either be rejected, or they will increase the frequency of the pulse and the general heat of the body; and thus, by wearing out its powers, exhaust that strength which they were intended to augment.

"Instead of this plan, the patient should take, two or three times in the day, a draught of infusion of columba, or some other light bitter, with the addition of a few grains of carbonate of ammonia.

"At the end of two or three weeks, it is to be expected that the stomach will have received an increase of strength; and then, instead of the volatile alkali, fifteen or twenty drops of tincture of muriate of iron, or of ammoniated iron, may be added to the draught.

"Sulphuric acid is a useful tonic in these cases, and may be given in an infusion or decoction of bark.

"A mixture of myrrh, steel, and alkali, with some aromatic water in some cases where the stomach will bear it, becomes a valuable remedy.

"Under a continuance of these or similar means, the patient's health will be gradually re-established.

"Care is to be taken to regulate the functions of the bowels until they have acquired sufficient strength to carry down their contents without assistance: for this purpose, the pil. ex aloë c. myrrh. or the pil. gamb. comp. of the last Pharmacopœia, are well enough adapted.

"It is advantageous in some cases, where the biliary secretion is sluggish, to exhibit occasionally a few grains of pil. hydr. at bed-time; and in the morning following a rhubarb draught. The strengthening remedies will produce a better effect when this is attended to than when it is omitted.

"A solution of sulphate of zinc or of alum may be thrown into the vagina by a syringe, three or four times a day; and if these should not be sufficiently powerful, such injections must be employed as possess a greater degree of astringency.

"Cold sea-bathing will be found very useful, when no symptoms are present forbidding it; the system, however, should have first rallied a little. Those patients who cannot bear the shock of a cold bath, will occasionally derive great advantage from a shower bath. The temperature of the water may be raised to sixty or seventy degrees, if the cold water does not agree with the patient."

We have thus presented our readers with a clear view and extended analysis of Mr. Clarke's volume. The matter speaks for itself, and the execution is highly creditable to the talents of our author.

